# Analysis of brain necrosis and secondary cancers after proton beam therapy for pediatric intracranial tumors: a single-center retrospective study

**DOI:** 10.3389/fonc.2025.1644839

**Published:** 2025-09-12

**Authors:** Masashi Mizumoto, Hiroko Fukushima, Yoshiko Oshiro, Takashi Saito, Ai Muroi, Yuni Yamaki, Sho Hosaka, Masako Inaba, Toshitaka Ishiguro, Masahiko Harada, Hikaru Niitsu, Toshiki Ishida, Taisuke Sumiya, Keiichiro Baba, Masatoshi Nakamura, Haruko Numajiri, Kei Nakai, Hideyuki Sakurai

**Affiliations:** ^1^ Department of Radiation Oncology, University of Tsukuba, Tsukuba, Japan; ^2^ Department of Pediatrics, University of Tsukuba Hospital, Tsukuba, Japan; ^3^ Department of Child Health, Institute of Medicine, University of Tsukuba, Tsukuba, Japan; ^4^ Department of Radiation Oncology, Tsukuba Medical Center Hospital, Tsukuba, Japan; ^5^ Department of Neurosurgery, Institute of Medicine, University of Tsukuba, Tsukuba, Japan; ^6^ Department of Radiology, University of Tsukuba Hospital, Tsukuba, Japan

**Keywords:** brain, brain necrosis, secondary cancer, proton beam therapy, particle therapy pediatric, radiation toxicity

## Abstract

**Background:**

Proton beam therapy (PBT) is increasingly used for pediatric intracranial tumors due to lower long-term radiation-associated toxicities. However, data on late adverse effects, particularly brain necrosis and intracranial secondary cancer, remain limited. The aim of this study is to evaluate the incidence of these events following PBT in pediatric patients treated at a single center.

**Procedure:**

We retrospectively reviewed the medical records of 189 patients under 20 years of age who received PBT for intracranial tumors between 1991 and 2023. Clinical information, irradiation parameters, concurrent chemotherapy, and follow-up outcomes were collected. Brain necrosis and intracranial secondary cancers were assessed based on events presenting with grade ≥2 clinical symptoms.

**Results:**

Among 151 patients with sufficient follow-up data (median follow-up: 41.7 months), two cases of brain necrosis (1.3%) and two cases of intracranial secondary cancer (1.3%) were identified. The 5-year cumulative incidence was 2.3% (95% CI: 0-5.4%) for brain necrosis and 2.7% (95% CI: 0-6.4%) for intracranial secondary cancer. These respective incidence rates were similar for patients followed for more than two years (n=94), and slightly higher at 2.7% and 3.1% for those receiving a total dose >50 Gy (n=134). Among patients treated with PBT alone (n=125), the incidence was 1.7% for brain necrosis and 3.6% for secondary malignancy.

**Conclusions:**

This single-center retrospective study shows a low incidence of brain necrosis and secondary malignancy following PBT for pediatric patients with intracranial tumors. These findings indicate a favorable long-term safety profile of PBT in this population.

## Introduction

Multidisciplinary treatment strategies, including surgery, chemotherapy, and radiotherapy (RT), have markedly improved outcomes for pediatric tumors (1, 2). As a result, the 5-year survival rate for children with cancer has reached approximately 80% (3). However, late adverse events, particularly those associated with RT, have become an important clinical issue. In pediatric intracranial tumors, such late complications include brain necrosis, secondary malignancies, hypopituitarism, and neurocognitive dysfunction (4–6). Proton beam therapy (PBT) has the potential to reduce the risk of these toxicities by minimizing radiation exposure to adjacent normal tissues, compared to conventional photon RT (7, 8). Although PBT has shown comparable efficacy to photon therapy when used with the same treatment protocols, clinical data on long-term adverse effects in pediatric patients remain limited. In particular, the incidence and nature of late adverse events such as intracranial secondary cancers and brain necrosis following PBT are not yet fully understood. To address this gap in knowledge, we conducted a single-center retrospective study to evaluate the frequency of these complications in pediatric patients with brain tumors treated with PBT.

## Methods

### Patients

From 1991 to 2023, a total of 189 patients under 20 years of age underwent PBT for intracranial tumors at our center. One radiation oncologist and one pediatric oncologist evaluated the following parameters in these cases: age, gender, total PBT dose, history of prior photon RT, combination with photon RT, irradiation method (e.g., local, whole-ventricle, craniospinal irradiation [CSI]), irradiation site, treatment outcome (alive or deceased), presence of brain necrosis, presence of intracranial secondary cancer, concurrent chemotherapy during irradiation, high-dose chemotherapy, intrathecal therapy, and concurrent methotrexate administration. The evaluation of all cases was independently conducted by one radiation oncologist and one pediatrician. When findings suggestive of secondary cancer or brain necrosis were identified, both physicians reviewed the case together. Brain necrosis was evaluated according to the CTCAE v5.0 criteria, and secondary intracranial cancers were identified based on pathological diagnosis or clinical diagnosis by radiologists. Symptoms of Grade 2 or higher were assessed using information obtained through written correspondence or telephone communication from referring centers or the patients themselves.

The typical treatment protocols were local irradiation of approximately 50–60 Gy (the total dose is expressed in GyE, which is calculated by multiplying the physical dose by an RBE of 1.1) for glioma and ependymoma, approximately 50–55 Gy following CSI of about 23.4-30.6 Gy for medulloblastoma, and approximately 50 Gy following whole-ventricle irradiation or CSI of about 30 Gy for embryonal brain tumors. Chemotherapy regimens and irradiation techniques and doses were generally consistent with those used in conventional photon RT.

### Statistical analysis

Intracranial secondary cancer incidence and brain necrosis incidence were calculated using SPSS v.29 (IBM, Armonk, NY, USA). The incidence rate was calculated using the start date of irradiation, the last follow-up date, and the date of death as the censoring date.

## Results

Of the 189 patients initially considered, 151 were included in the analysis. Thirty eight patients were excluded due to lack of availability of post-irradiation follow-up data, irradiation dates that were too remote to ensure data integrity, or treatment protocols that did not meet the study standards. The characteristics of the 151 patients are summarized in **Table 1**. The median age was 8.3 years (range, 0–19 years) and 90 patients (59.6%) were male. Twenty-six patients received a combination of PBT and photon RT. Proton beam therapy was performed using local (63.6%) or whole ventricle irradiation (20.5%). Craniospinal irradiation was administered in 11.3% of cases, and whole-brain irradiation in 4.6%. Patient characteristics and treatment details, including age distribution, tumor types, irradiation methods (e.g., local, whole-ventricle, craniospinal irradiation), and chemotherapy regimens, are summarized in **Table 1**. Photon therapy was combined in cases requiring craniospinal or whole-brain irradiation. The tumor types was germ cell tumor (n=41), ependymoma (n=31), brainstem tumor (n=22), glioma (n=19), medulloblastoma (n=11), atypical teratoid/rhabdoid tumor (AT/RT; n=10), primitive neuroectodermal tumor (PNET; n=4), pineal tumor (n=3), meningioma (n=3), craniopharyngioma (n=2), and other tumors including pathologically unclassified brain tumors (n=5).

**Table 1:** Patient background.

Factors	All patients	2-year follow-up	50GyE or more	PBT alone
N	151	94	134	125
Follow-up (months)				
median	41.7	71.6	37.5	33.7
Range	0-168.2	25.1-168.2	0-168.2	0-161.2
Age				
Median	8.3	8.8	6.9	7.8
Range	0.6-19.8	1.0-19.5	1.0-19.8	1.0-19.8
Gender				
male	90 (59.6)	62 (66.0)	78 (58.2)	75 (60.0)
Total dose				
median	54.0	54.0	54.0	54.0
range	23.4-73.5	23.4-61.6	50-73.5	23.4-73.5
Irradiation method				
Local	96 (63.6)	45 (47.9)	95 (70.9)	94 (75.2)
whole ventricle	31 (20.5)	26 (27.7)	16 (11.9)	31 (24.8)
whole brain	7 (4.6)	7 (7.4)	7 (5.2)	0
CSI	17 (11.3)	16 (17.0)	16 (11.9)	0
Tumor Type				
germ cell tumor	41 (27.2)	36 (38.3)	26 (19.4)	29 (23.2)
ependymoma	31 (20.5)	21 (22.3)	31 (25.4)	31 (24.8)
brain stem tumor	22 (14.6)	2 (2.1)	21 (15.7)	21 (16.8)
Glioma	19 (12.6)	8 (8.5)	19 (14.2)	18 (14.4)
Medulloblastoma	11 (7.3)	10 (10.6)	11 (8.2)	4 (3.2)
AT/RT	10 (6.6)	6 (6.4)	9 (6.7)	8 (6.4)
PNET	4 (2.6)	2 (2.1)	4 (3.0)	3 (2.4)
pineal tumor	3 (2.0)	1 (1.1)	3 (2.2)	3 (2.4)
meningioma	3 (2.0)	3 (3.2)	3 (2.2)	3 (2.4)
craniopharyngioma	2 (1.3)	1 (1.1)	2 (1.5)	2 (1.6)
Others	5 (3.3)	4 (4.3)	5 (3.7)	3 (2.4)
Chemotherapy				
concurrent	74 (49.0)	52 (51.5)	60 (44.8)	52 (41.6)
high dose Interval (days)	16 (10.6) 52 (28–957)	10 (10.6) 52 (37–388)	15 (11.2) 57 (37–957)	12 (9.6) 52 (37–957)
intramedullary	30 (19.9)	22 (23.4)	28 (20.9)	20 (16.0)
high dose MTX	12 (7.9)	8(8.5)	12 (9.0)	12 (9.6)

2-year follow-up: Group followed for more than 2 years, 50GyE or more: Groups irradiated with 50 GyE or more, PBT alone: Group treated with proton therapy alone. CSL, Craniospinal irradiation; AT/RT, Atypical teratoid/rhabdoid tumor; MTX, methotrexate.

Interval (days): This interval was defined as the number of days from autologous hematopoietic stem cell transplantation to the initiation of the nearest proton therapy session.

Concurrent chemotherapy was administered in 74 patients. In addition, high-dose chemotherapy was given in 16 patients, intrathecal chemotherapy in 30, and high-dose methotrexate in 12. These treatments may have overlapped among patients. The median interval between high-dose chemotherapy and proton therapy was 52 days (range, 28–957 days). This interval was defined as the number of days from autologous hematopoietic stem cell transplantation to the initiation of the nearest proton therapy session.

The chemotherapy regimens were documented in most cases, with the most common being CARE-based protocols (n=23, 87.0% with concurrent chemotherapy), ICE-based regimens (n=14, 92.9%), and TMZ-based regimens (n=16, 87.5%). Other regimens (n=73) included a variety of institutional or tumor-specific combinations. The regimens were selected based on tumor histology, clinical risk stratification, and institutional preferences. RT strategies included local irradiation (n=96), CSI (n=17), whole-brain irradiation (n=7), and whole-ventricular irradiation (n=31). The median total dose was 54.0 Gy (range, 23.4-73.5 Gy), with 134 patients receiving a dose >50 Gy. Details of the treatment methods for each patient are provided in the supplement, along with the corresponding references documenting the established treatment protocols (9–23).

The median follow-up period for all patients was 41.7 months (range, 0-168.2 months). Brain necrosis was observed in two patients (**Table 2**). Patient 1 underwent reirradiation with PBT for local recurrence (cumulative dose: 113 Gy) and developed mild symptomatic brain necrosis 34.7 months after the initial irradiation. Patient 2 developed a small, asymptomatic contrast-enhancing lesion within the irradiated area 25.1 months after PBT. Although minor symptoms occasionally occurred thereafter, these were manageable with medical treatment. Secondary malignancies occurred in two patients. Patient 4 developed glioblastoma, while Patient 3 presented with a brainstem tumor of unconfirmed pathology. Both patients died of these secondary malignancies.

**Table 2:** Brain necrosis or intracranial secondary cancer cases.

Patient number	Event	Age	Gender	Total dose	Photon	Tumor	Method	Chemotherapy	Time to event (months)
1	Necrosis	6.2	M	54.0	None	EPN	Local	Concurrent, methotrexate intrathecal therapy	34.7
2	Necrosis	10.5	F	54.0	Yes	MB	CSI	Concurrent	25.1
3	2^nd^ cancer	1.8	M	50.4	None	EPN	Local	None	39.1
4	2^nd^ cancer	3.3	M	59.4	None	EPN	Local	None	44.3

M, male; F, female; Total dose, total irradiation dose; Photon, combined photon radiotherapy; Local, local irradiation; CSI, craniospinal irradiation; EPN, ependymoma; MB, medulloblastoma.

**Figure 1:**
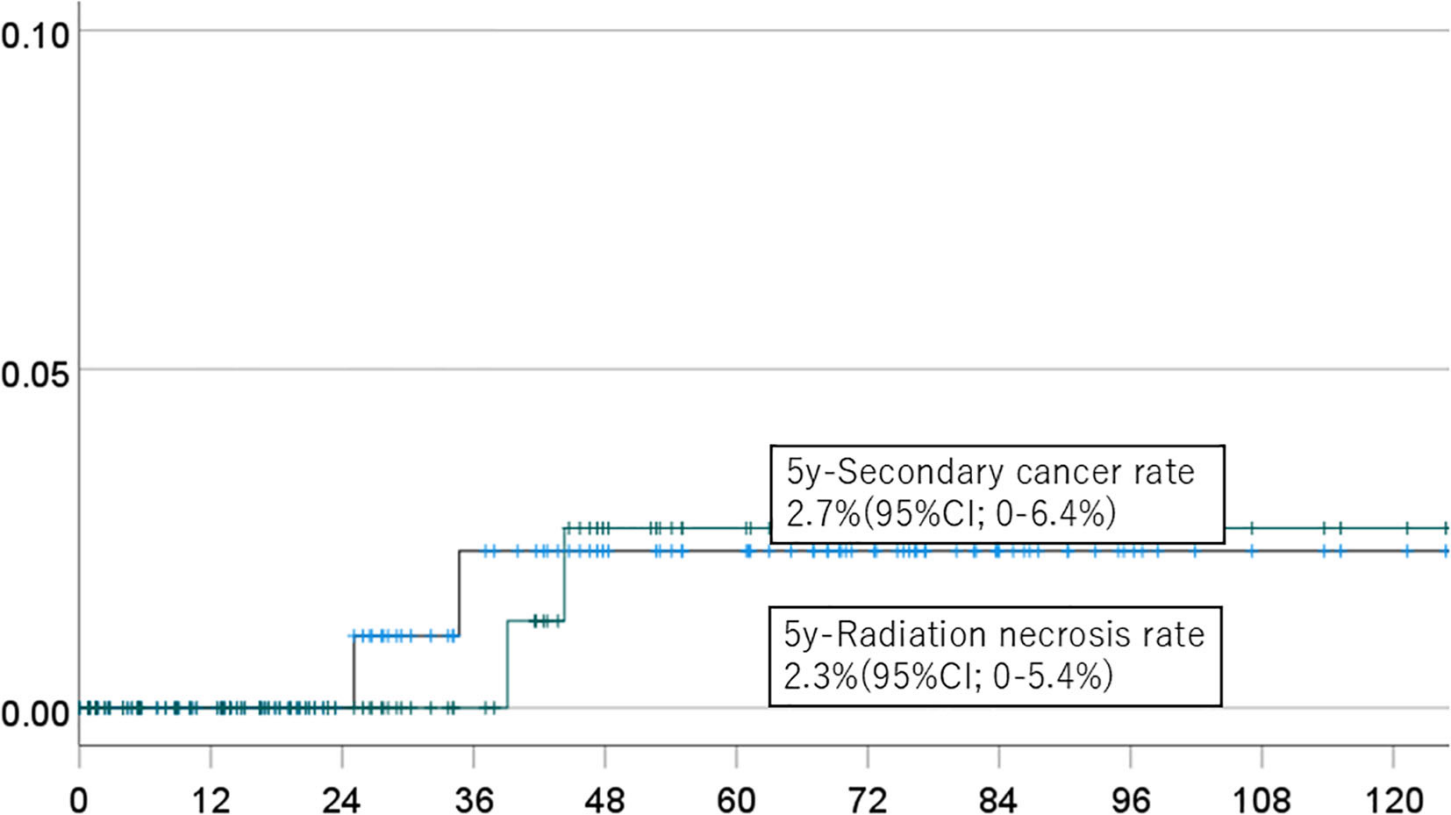
Incidence of brain necrosis (solid line) and intracranial secondary cancer (dotted line) in all patients.

**Figure 2:**
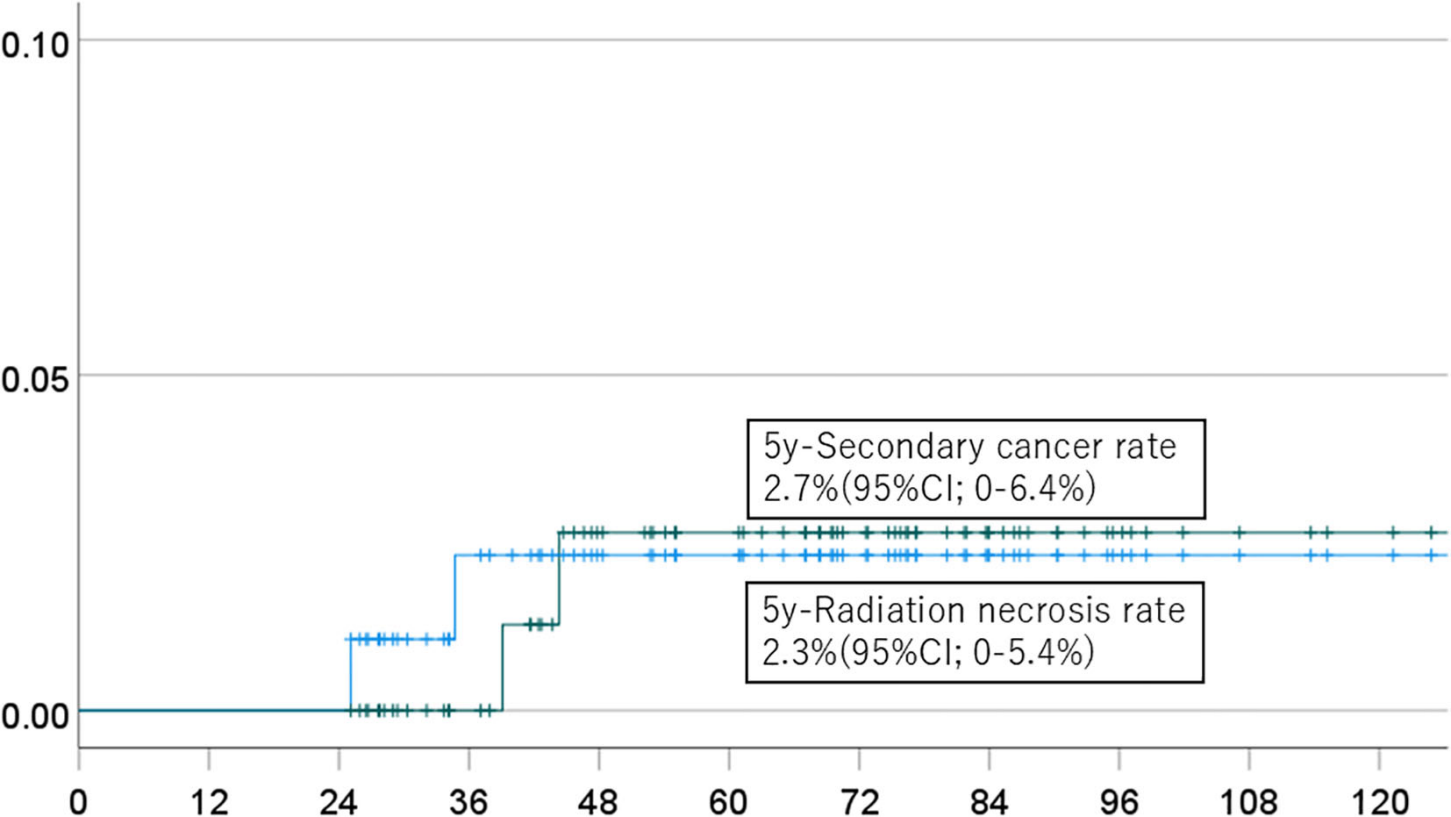
Incidence of brain necrosis (solid line) and intracranial secondary cancer (dotted line) in patients followed for more than 2 years.

**Figure 3:**
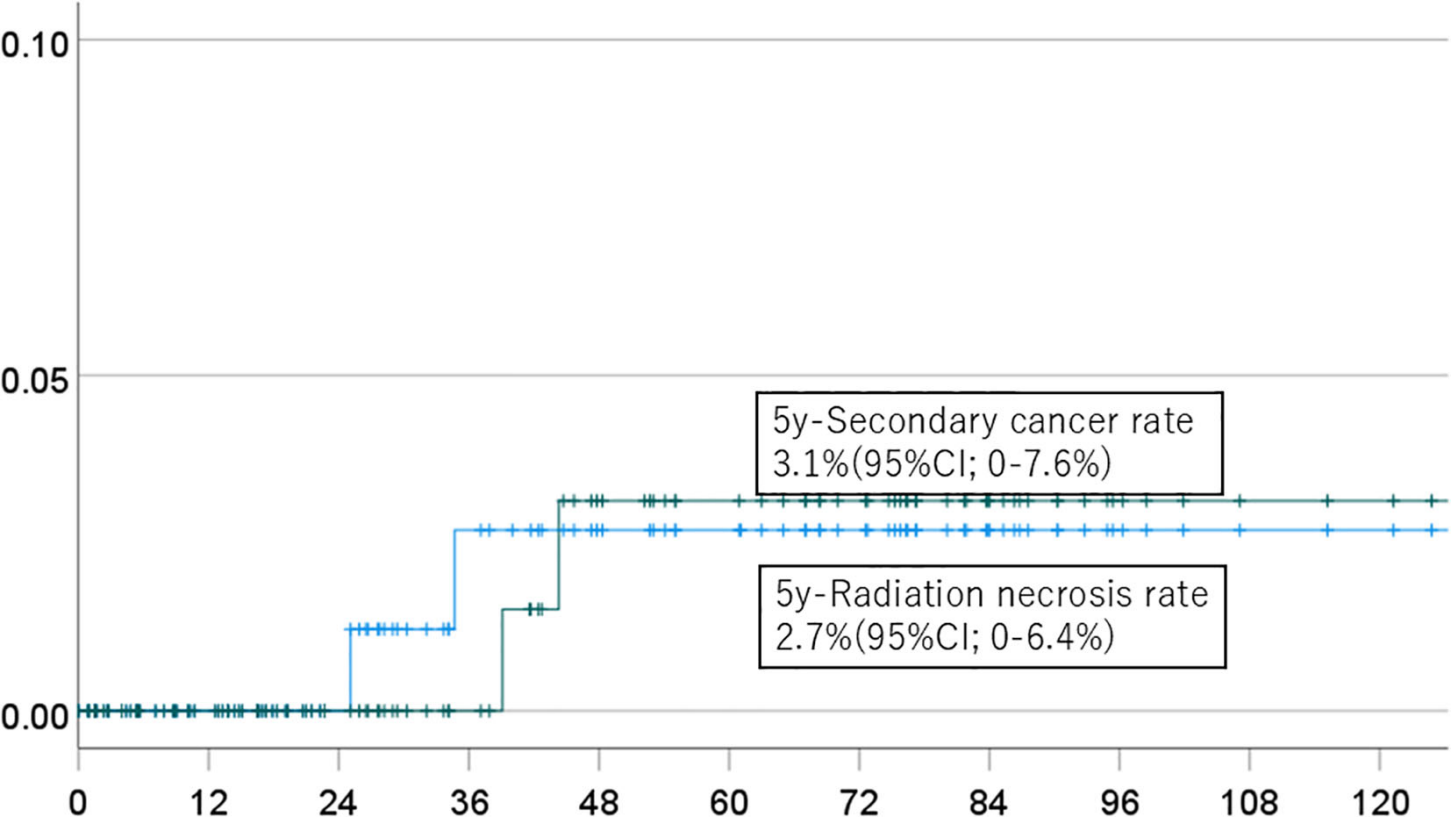
Incidence of brain necrosis (solid line) and intracranial secondary cancer (dotted line) in patients who received a dose of 50 Gy or more.

**Figure 4:**
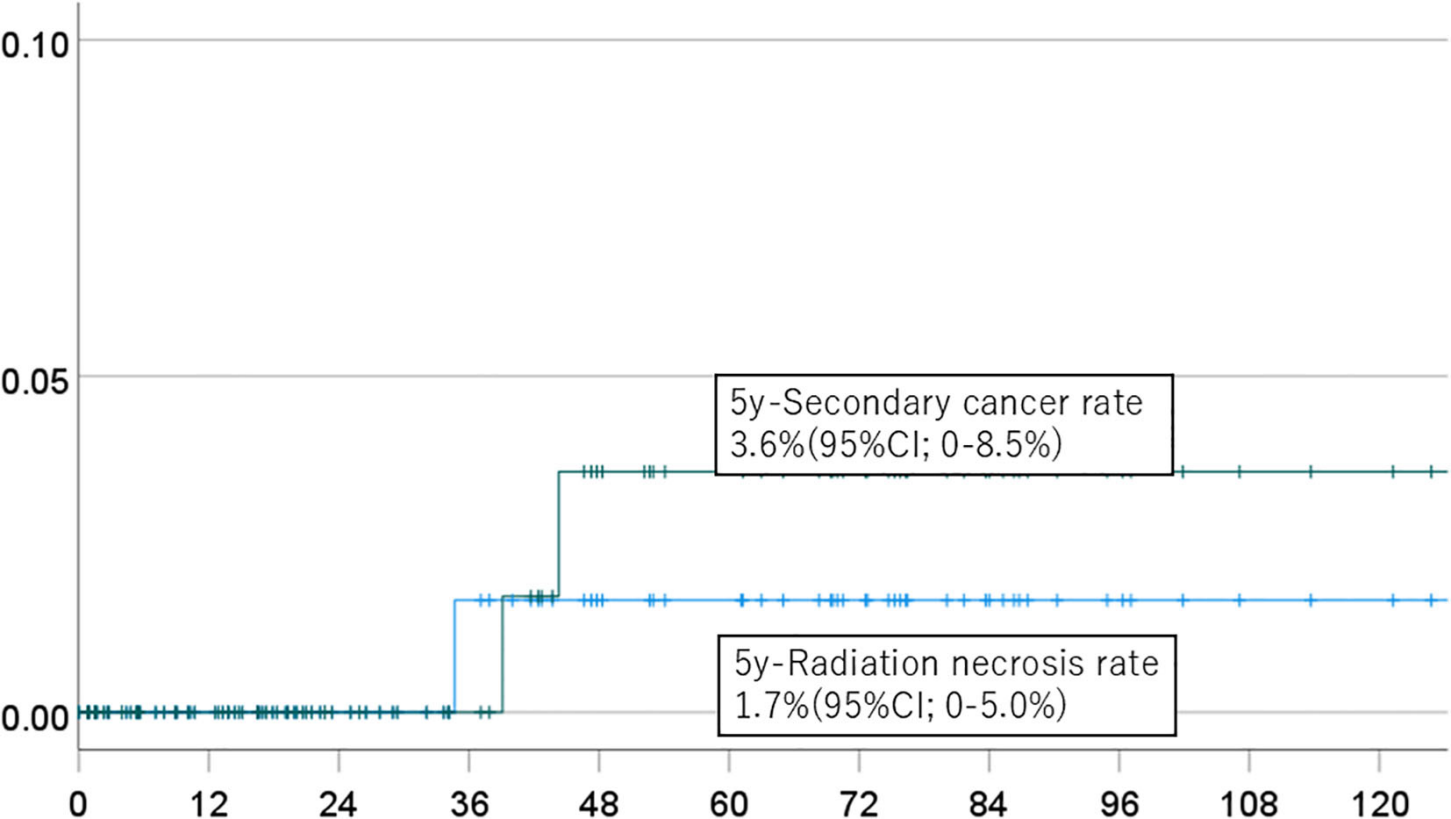
Incidence of brain necrosis (solid line) and intracranial secondary cancer (dotted line) in patients who received proton beam therapy alone.

The 5-year cumulative incidence rates of brain necrosis and secondary malignancy for all patients (n=151) were 2.3% (95% CI, 0-5.4%) and 2.7% (95% CI, 0-6.4%), respectively (**Figure 1**). These rates were 2.3% (95% CI, 0-5.4%) and 2.7% (95% CI, 0-6.4%) in patients (n=94) followed for more than two years (**Figure 2**); 2.7% (95% CI, 0-6.4%) and 3.1% (95% CI, 0-7.6%) in patients (n=134) with a total dose >50 Gy (**Figure 3**); and 1.7% (95% CI, 0-5.0%) and 3.6% (95% CI, 0-8.5%) in patients (n=125) treated with PBT alone (**Figure 4**). Radiation necrosis was observed in one patient who underwent reirradiation, suggesting a potential increased risk in this subgroup. In contrast, RN occurred in only one patient among those who received initial irradiation. Additionally, patients who received a total dose exceeding 50 Gy showed slightly higher incidence rates of RN and secondary malignancy (2.7% and 3.1%, respectively) compared to those receiving ≤50 Gy. Given the limited number of events, no definitive statistical analysis was conducted; however, these trends suggest that reirradiation and higher total dose may be associated with an increased risk of late adverse events.

## Discussion

Since both proton and photon beams are categorized as low linear energy transfer (low-LET) radiation, their therapeutic effects are considered equivalent when the same area is irradiated with comparable dose fractions (7). Indeed, Eaton et al. found no significant difference in overall survival or disease control between photon and proton therapies in pediatric patients with medulloblastoma (24). However, due to the presence of the Bragg peak, proton beams allow for superior dose localization compared to photon beams. This enables a reduction in the volume of normal tissue exposed to low-dose radiation, and multiple dosimetric studies have shown the superiority of PBT in this regard (25–28). This is particularly relevant in pediatric patients, in whom cognitive impairment is a major concern and the mean dose to the brain may be associated with long-term neurocognitive outcomes (6). The dose to tissues adjacent to the high-dose target volume in PBT can be comparable to that of photon RT, and the associated risk of local toxicities such as brain necrosis remains. However, few studies have examined the incidence of brain necrosis following RT in pediatric patients.

In a study at St. Jude Children’s Research Hospital, brainstem necrosis occurred in 3.7% of patients within five years after photon RT, and incidence rates in other trials have ranged from 2.2% to 8.6% (29–31). For PBT, Indelicato et al. analyzed 313 patients who received ≥50.4 Gy to the brainstem and found a 2-year incidence of late adverse events of 3.8%, including a 2.1% rate of Grade ≥3 brainstem injury (32). A meta-analysis by Alrasheed et al. found a 1.5% incidence of Grade 2 brainstem toxicity following PBT for pediatric brain tumors (33). To further contextualize the incidence of brain necrosis observed in our cohort, we summarized representative studies reporting brain necrosis rates in pediatric brain tumor patients treated with proton or photon radiotherapy (**Table 3**). The risk of brain necrosis is influenced by multiple factors, including radiation dose, irradiated volume, age at the time of treatment, tumor location in the infratentorial region, prior surgical interventions, and use of high-dose chemotherapy (32, 37). Additionally, pediatric brain tumors are rare and treatment protocols vary, making it challenging to establish high-level evidence. In the present study, we investigated the incidence of brain necrosis following PBT in pediatric brain tumor cases. Brain necrosis occurred in two patients, one of whom had undergone reirradiation before the onset of necrosis. Given that reirradiation is a known significant risk factor for radiation necrosis (36), exclusion of these cases from the analysis could have potentially reduced the observed incidence. However, we deliberately chose not to exclude patients who underwent reirradiation with proton therapy in order to avoid selection bias and to present an unbiased representation of real-world clinical practice. Excluding such cases might have been interpreted as selectively removing unfavorable data, which could compromise the transparency and generalizability of our findings. Therefore, we included all patients except those who had undergone prior photon radiotherapy before proton beam therapy, ensuring that the reported incidence rates reflect the actual clinical outcomes of our institution’s PBT practice. In this study, the number of events for both secondary malignancies and brain necrosis was limited, precluding a multivariate analysis to identify patient-specific risk factors. To address this limitation, we are currently collaborating with the two largest pediatric proton therapy centers in Japan and the leading pediatric proton facility in China to conduct an expanded analysis with a larger patient cohort. Additionally, we recognize that the relatively short median follow-up period limits the ability to assess very late-onset adverse events such as secondary malignancies. Continued long-term follow-up and additional analyses will be essential to provide a more comprehensive understanding of these risks.

**Table 3:** Comparison of cerebral radiation necrosis incidence in pediatric brain tumor patients.

Author, Year	Cohort, Tumor	Methods median dose	No. of PT	Median follow-up	Brain necrosis (%)
Drezner 2016 (34)	Pediatric CNS tumors	Proton and photon radiotherapy (summary of 9 studies)	806	33 months (24–52)	4.6% (37/806)
Indelicato 2014 (32)	Pediatric brain/skull base tumors	Proton 54GyRBE	313	24 months	3.5% (11/313)
Murphy 2012 (31)	CNS embryonal tumors	Photon 55.8Gy	236	52 months	3.7% (8/236)
Orukari 2022 (35)	Posterior fossa tumors	Proton 54GyRBE	58	more than 1 year	0%
Ajithkumar 2024 (36)	Pediatric CNS tumors	Re-irradiation 111.4 Gy (summary of 17 studies)	449	1.6 years	4.9% (95%CI, 3.1-7.3%)
Current Study	Pediatric CNS tumors	Proton 54GyRBE	151	41.7 months	1.3% (2/151)

PT, Patient.

The estimated 5-year incidence of Grade ≥2 brain necrosis ranged from 1.7% to 2.7%, suggesting that the risk of brain necrosis in our cohort was relatively low. This study has several limitations. First, the number of events was small, which may limit the statistical power to detect rare adverse outcomes. Second, the median follow-up duration of 41.7 months may not be sufficient to fully capture very late adverse effects such as secondary malignancy, which can develop over a longer time frame. Third, in some cases, long-term follow-up and imaging were conducted at referring institutions, and we could not centrally confirm all radiological findings. Therefore, the true incidence of late toxicities might be underestimated. Thus, PBT for pediatric intracranial tumors delivered using protocols comparable to those for conventional photon RT appears to have no increased risk of secondary malignancy or radiation-induced brain necrosis.

Secondary malignancy remains a significant concern following cranial irradiation in pediatric patients. Sethi et al. reported 10-year incidence rates of secondary malignancies in the irradiated field of 14% for photon RT and 0% for PBT in a cohort of 84 patients with retinoblastoma (38). Zhang et al. used in silico modeling of 17 CSI cases of medulloblastoma to estimate a lifetime risk ratio of 0.10 to 0.22 for secondary malignancy associated with PBT *vs*. photon RT (39). Similarly, Yoon et al. analyzed 10 CSI cases and found that the risk of secondary malignancy with photon RT was at least five times greater than that associated with PBT (40). In a comparison of 558 patients (including 44 children) treated with PBT to a matched cohort treated with photon RT selected from the U.S. SEER database, Chung et al. found a secondary malignancy incidence of 5.2% after PBT and 7.5% after photon RT, with a 10-year cumulative incidence of 5.4% *vs*. 8.6% and a hazard ratio of 0.52 (p = 0.009) (41). In our previous nationwide survey in Japan, the 10-year incidence of secondary malignancy following pediatric PBT was 5% (42).

In the current study, secondary malignancy occurred in 2 of 151 patients, giving a 5-year cumulative incidence of 2.7 to 3.6%. The follow-up period was relatively short and the number of secondary malignancy cases was low, which prevents a definitive conclusion, but our findings do not suggest a higher incidence compared with previous reports. Thus, PBT for pediatric intracranial tumors delivered using protocols comparable to those for conventional photon RT appears to have no increased risk of secondary malignancy or radiation-induced brain necrosis.

## Data Availability

The original contributions presented in the study are included in the article/**Supplementary Material**. Further inquiries can be directed to the corresponding author.
